# Differential diagnosis of uncommon prostate diseases: combining mpMRI and clinical information

**DOI:** 10.1186/s13244-021-01024-3

**Published:** 2021-06-16

**Authors:** Chao Han, Lina Zhu, Xiang Liu, Shuai Ma, Yi Liu, Xiaoying Wang

**Affiliations:** 1 Department of Radiology, Peking University First Hospital, No. 8 Xishiku Street, Xicheng District, Beijing, 100034 China; 2 Department of Radiology, The First Affiliated Hospital of Zhengzhou University, No.1 Jianshe Road, ZhengzhouHenan Province, 450052 China

**Keywords:** Prostate, Magnetic resonance imaging, Uncommon prostatic diseases

## Abstract

The differential diagnosis of abnormalities in the prostate is broad, covering common (acinar adenocarcinoma, benign prostatic hyperplasia, chronic prostatitis, hemorrhage, cysts, calcifications, atrophy and fibrosis) and less common conditions (tumors other than acinar adenocarcinoma, granulomatous prostatitis containing tuberculosis, abscesses and other conditions, and idiopathic disorders such as amyloidosis and exophytic benign prostatic hyperplasia). Recent advances in magnetic resonance imaging (MRI) of the prostate gland and imaging guidelines, such as the Prostate Imaging Reporting and Data System version 2.1 (PI-RADS v2.1), have dramatically improved the ability to distinguish common abnormalities, especially the ability to detect clinically significant prostate cancer (csPCa). Overlap can exist in the clinical history and imaging features associated with various common/uncommon prostate abnormalities, and biopsy is often required but is invasive. Prostate abnormalities can be divided into two categories: category 1, diseases for which PI-RADS scores are suitable for use, and category 2, diseases for which PI-RADS scores are unsuitable for use. Radiologists must have an intimate knowledge of other diseases, especially uncommon conditions. Past relevant history, symptoms, age, serum prostate-specific antigen (PSA) levels, MRI manifestations, and the applicability of the PI-RADS assessment should be considered when diagnosing prostate abnormalities.

## Key Points

- Prostate abnormalities can be divided into two categories, PI-RADS suitable and PI-RADS unsuitable.
- Radiologists must have an intimate knowledge of uncommon prostate abnormalities.
- Granulomatous prostatitis adds a diagnostic challenge as it usually mimics PCa radiologically.
- Most sarcomas appear as well-circumscribed masses with a complete or incomplete pseudocapsule.

## Background

Diseases of the prostate make up a large proportion of male urologic diseases. In the clinic, individuals usually visit the hospital with overlapping symptoms of prostatic diseases or elevated serum prostate-specific antigen (PSA) levels. Currently, advances in multiparametric magnetic resonance imaging (mpMRI) of the prostate gland and the Prostate Imaging Reporting and Data System version 2.1 (PI-RADS v2.1) have remarkably improved the ability to detect and stage clinically significant prostate cancer (csPCa) [1–4].

PI-RADS v2.1 suggests the use of the mpMRI protocol, including T2-weighted imaging (T2WI), diffusion-weighted imaging (DWI), apparent diffusion coefficient (ADC) maps and dynamic contrast-enhanced (DCE) imaging, to provide both imaging and functional information of the prostate. In addition, radiologists use PI-RADS v2.1 assessment categories, a 5-point scale from PI-RADS 1 to5, to express the probability of the presence of csPCa for each lesion in the prostate gland [3]. Therefore PI-RADS v2.1 is widely applied in the clinic to distinguish csPCa from benign prostatic hyperplasia (BPH), chronic prostatitis, or other common prostatic diseases. However, unusual diseases involving the prostate, such as uncommon prostatic neoplasms, are classified into multiple classes. As a result of their relative rarity and diverse of imaging presentations [5], many radiologists might not be familiar with their characteristics or situations in which the use of the PI-RADS assessment is appropriate.

Therefore, in this article, prostatic diseases are divided into two categories: category 1, diseases for which the PI-RADS assessment is suitable for use, and category 2, diseases for which PI-RADS assessment is not suitable for use. The characteristics of common prostatic diseases are simply reviewed, followed by a focus on the characteristics of unusual prostatic diseases. In addition, we summarize the clinical MRI diagnostic workflow for prostatic diseases.

## Classification of prostatic diseases

Common diseases of the prostate include acinar adenocarcinoma, BPH, chronic prostatitis, hemorrhage, cysts, calcifications, atrophy and fibrosis. Uncommon diseases of the prostate include tumors other than acinar adenocarcinoma, granulomatous prostatitis containing tuberculosis, abscesses and so on, and idiopathic disorders such as amyloidosis and exophytic BPH.

Many conditions that yield abnormal signals within the prostate, including hemorrhage, cysts, calcifications, atrophy and fibrosis, are benign and highly recognizable on mpMRI [3]. In addition to these benign signal abnormalities and based on the applicability of PI-RADS assessment, we divide other focal signal abnormalities involving the prostate into two categories according to the patient’s age, serum PSA level, symptoms and mpMRI findings: category 1, diseases for which the PI-RADS assessment is suitable for use, and category 2, diseases for which the PI-RADS assessment is not suitable for use. Category 1 includes prostate cancer (PCa), typical BPH in the transitional zone (TZ), and some types of prostatitis/granulomatous prostatitis, which overlap in terms of clinical and mpMRI findings, while category 2 includes tumors except for PCa, exophytic BPH nodules, and some types of granulomatous prostatitis (abscesses and tuberculosis), for which PCa may be excluded according to the clinical and MRI findings.

## Common prostatic diseases other than typical benign lesions

Common prostatic diseases other than typical benign lesions, such as acinar adenocarcinoma, BPH and prostatitis, are usually inert or chronic, with varying degrees of elevated serum PSA levels or disturbing symptoms such as lower urinary tract symptoms (LUTS), and interfere with the quality of life as a long-term problem for males, particularly elderly males, as BPH and prostate cancer are age-related conditions [6]. PSAs are proteinases produced mainly in the epithelial cells of the prostate [7]. When various factors cause destruction of the epithelial cells or the blood-epithelial barrier, a substantial increase in PSA secretion from tumor cells, or increasing entry of PSAs into the blood, serum PSA levels are increased.

PCa is one of the most common group of malignancies occurring in the male population after lung cancer [8], among which acinar adenocarcinoma is the most common malignancy observed. On MRI, csPCa presents with homogeneous and moderate hypointensity on T2WI, hyperintensity on high b-value DWI, a low ADC, and early enhancement, without capsules and easily forming extraprostatic extensions (EPEs). These findings result in a PI-RADS 4–5 classification.

BPH is the fifth most prevalent non-cancer-related disorder among 50-year-old or elderly men [9]. By the age of 60, the prevalence of BPH is 50%, and it increases to 80% by the age of 80. BPH tissue produces PSA and primarily arises in the TZ. BPH consists of glandular hyperplasia and stromal hyperplasia. Predominantly glandular BPH nodules exhibit moderate hyperintensity on T2WI with hypointense capsules, while predominantly stromal nodules present hypointensity on T2WI. A mixture of stromal and glandular hyperplasia may appear as band-like areas and/or encapsulated round nodules with circumscribed or encapsulated margins. Many BPH nodules display a mixture of signal intensities on T2WI and may be remarkably enhanced on DCE imaging [3]. The PI-RADS assessment is suitable for use and PI-RADS scores of 1–2 may be assigned to typical BPH.

Chronic prostatitis is often subclinical and presents as an immune infiltrate. On T2WI and ADC maps, chronic prostatitis often presents with decreased signals that are band-like, wedge-shaped, or diffuse in the peripheral zone (PZ). On DCE imaging, they may exhibit early enhancement. The PI-RADS assessment is suitable for use and a PI-RADS score of 2 may be assigned to typical chronic prostatitis.

## Uncommon prostatic diseases

### BPH nodules outside the TZ

Although BPH develops in the TZ, hyperplastic prostatic nodules may also be found in the PZ or central zone (CZ). Peripheral hyperplastic nodules comprise up to 18.5% of prostate specimens with palpable and/or ultrasonographically hypoechoic focal lesions [10], and less than 10.0% of ultrasonographically hypoechoic focal nodules located in the PZ of the prostate are histologically confirmed as BPH [11, 12].

Some authors believe that BPH arises in the TZ, but a few BPH nodules can be exophytic or extrude into the PZ or CZ. However, Jie Tang et al. indicated that some BPH nodules in the PZ originate from the PZ, and the formation of these nodules may be modulated in a different manner than those that form in the TZ because the relative expression of proteins involved in the regulation of prostate proliferation and apoptosis differs between PZ and TZ hyperplastic nodules [13].

**Fig. 1:**
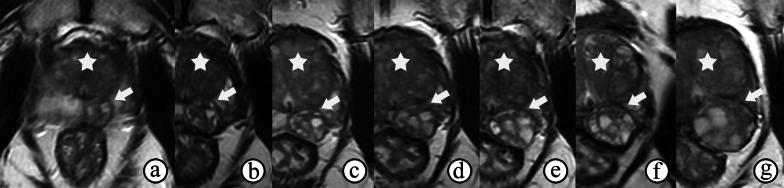
BPH in a 66-year-old man with an elevated serum PSA level fluctuating from 4 to 10 ng/mL. **a–g** Axial T2-weighted images captured in Jan 2010 (size of 1.3 cm), May 2012, Mar 2013, Dec 2013, Feb 2015, May 2015, and Jul 2019 (size of 2.6 cm) show a gradually increasing nodule with heterogeneous hyperintensity and a well-defined capsule in the left peripheral zone (arrow), the size of which increases with the progress of BPH in the TZ (☆)

PZ and TZ hyperplastic nodules are similar regarding patient age, PSA level, prostate volume and nodule size [13, 14]. BPH nodules outside the TZ are usually well-circumscribed lesions with an ovoid or round shape and a smooth surface on MRI, and the size of the nodule may also increase with the progress of BPH in the TZ (Fig. 1). In this situation, the PI-RADS assessment is not suitable for use.

### Granulomatous prostatitis

Recently, radiologists’ knowledge of granulomatous prostatitis has improved because of an increase in the prevalence of the disease due to an increase in the implementation of various surgical interventions, such as transurethral resection of prostate (TURP) and needle biopsy procedures. Granulomatous prostatitis is an unusual and benign inflammatory condition of the prostate classified based on etiology and histopathology into the following types: idiopathic (nonspecific), infective, iatrogenic (after surgical operation), malakoplakia, and cases associated with systemic granulomatous diseases and allergy [15]. Among these conditions, nonspecific granulomatous prostatitis is the most common accounting for 45–77.7% of cases, for which autoimmunity has been recognized as a key factor in the pathogenesis [16, 17]. In total, granulomatous prostatitis accounts for approximately 3.3% of all benign inflammatory lesions of the prostate and less than 2% of all prostatic specimens, including prostatic biopsies, TURP chips, and radical prostatectomies [15, 17].

Clinically, granulomatous prostatitis may present with symptoms including irritation and LUTS, with or without hematuria, fever and chills, and is frequently associated with transiently increased serum PSA levels, which may decrease with the resolution of the inflammation [18]. The age of onset ranges from 18 to 86 years, with a mean age of 70 years [15, 17].

**Fig. 2:**
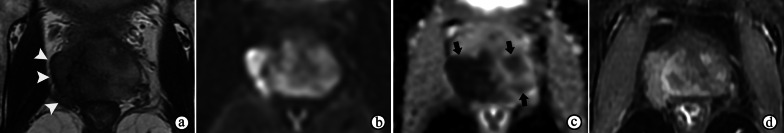
Nonspecific granulomatous prostatitis in a 57-year-old male who experienced frequency, urgency and burning micturition for 1 month, with a serum PSA level of 18.67 ng/mL transiting to 5.04 ng/mL. **a–c** T2WI, DWI at a b-value of 1400 s/mm^2^ and ADC maps show a diffusely and multifocally low signal intensity in the surrounding parenchyma on T2WI (**a**), a high signal intensity on DWI (**b**) and low ADC values (arrows on **c** in the prostate, with extraprostatic extension (arrowheads on **a**). (**d**) DCE imaging shows diffuse and multifocal areas of enhancement. The abnormal appearances of the prostate in the images shown in **a–d** result in a PI-RADS score of 5

**Fig. 3:**
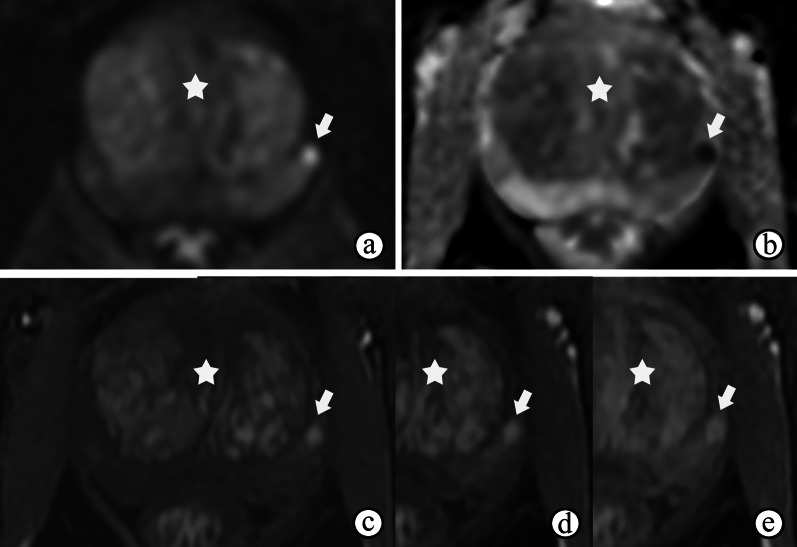
Nonspecific granulomatous prostatitis in a 57-year-old male with dysuria and urinary retention for 1 month and a serum PSA level of 13.22 ng/mL. **a**, **b** DWI at a b-value of 1400 s/mm^2^ and ADC maps show a focal nodule with obviously restricted diffusion (arrow) in the left peripheral zone of the prostate. **c–e** DCE imaging shows early and prolonged enhancement of the nodule (arrow). The appearances of the nodule in **a–e** result in a PI-RADS score of 4. Note the prostatic hyperplasia in the TZ (☆), which may have caused the symptoms of dysuria and urinary retention

**Fig. 4:**
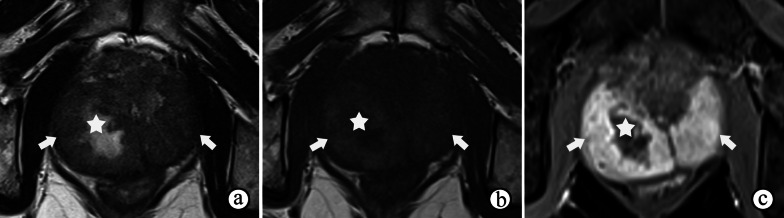
Diffuse granulomatous prostatitis in a 67-year-old male. **a** T2WI shows diffuse swelling of bilateral peripheral zones and the signal intensity is diffusely decreased (arrow). **b** T1WI shows a diffusely increased signal intensity in the bilateral PZs (arrow). **c** DCE imaging shows a rapid enhancement of bilateral PZs (arrow). Note the central necrosis in the lesion (☆)

On mpMRI, granulomatous prostatitis strongly mimics PCa regardless of whether it is diffuse (Fig. 2) or focused (Fig. 3), with hypointensity on T2WI and obviously restricted diffusion on DWI and ADC maps, and presents earlier or contemporaneous with the enhancement of adjacent normal prostatic tissues on DCE imaging [19] (Figs. 2d and 3c). In addition, capsular bulges or capsular irregularities suspected of early extracapsular extension can also be observed (Fig. 2). The majority of lesions of granulomatous prostatitis confirmed on fusion-targeted biopsies are assigned a PI-RADS score of 4 or 5 [20]. Interestingly, some cases of diffuse granulomatous prostatitis may present with hyperintense signals on T1WI [21] (Fig. 4b). The diagnosis of granulomatous prostatitis is also challenging because it usually mimics PCa both clinically and radiologically, and the PI-RADS assessment may be used unavoidably; hence the diagnosis is only possible by performing a histopathological examination.

#### Prostate abscess

Central necrosis can occur in several cases of granulomatous prostatitis. Abscesses may form by the combined effect of central inflammatory cells, proteins, cellular debris, and water molecules bound to macromolecules [22] and are more common in infective than in other types of granulomatous prostatitis. Infective granulomatous prostatitis can be caused by bacteria (such as *E. coli*, *Staphylococcus species*, and *Mycobacterium tuberculosis*), viruses (*herpes zoster*), fungi (*Cryptococcus*, *Candida*, *Aspergillus*), or *Treponema pallidum* [23, 24]. Typical signs and symptoms may include fever, chills, urinary frequency and urgency, suprapubic pain, dysuria, and hematuria. Urine examination usually reveals pus cells.

**Fig. 5:**
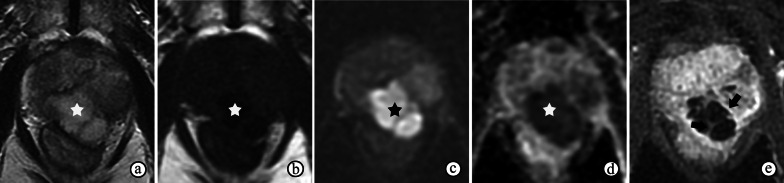
Prostate abscess in a 68-year-old male with dysuria for 2 months and a serum PSA level of 9.50 ng/mL, 16.9 white blood cells/high power field in urine and 1.7 epithelial cells /high power field in urine. **a, b** T2- and T1-weighted imaging show a mass with high T2 signal intensity and low T1 signal intensity (☆) in the PZ compressing the rectum. **c, d** The interior of the mass shows a very high signal intensity on DWI at a b-value of 800 s/mm^2^ and a very low signal intensity on the ADC maps (☆). **e** DCE imaging shows enhancement of the rim and separations in the middle (arrow)

DWI and ADC maps demonstrate the highly viscous internal contents of abscesses, which are surrounded by granulomatous infiltration that appears as low T2 signal intensity, restricted diffusion, and moderate enhancement. The features on MRI are typical (Fig. 5); the interior of the central necrosis presents with hyperintensity on T2WI, hyperintensity on high b-value DWI, and low ADC, and the exterior demonstrates rim enhancement [16, 23]. PI-RADS may not be applicable in these situations. Nevertheless, MRI is useful for evaluating the prevalence of occult caseous abscesses and for follow-up after treatment.

#### Prostate tuberculosis

**Fig. 6:**
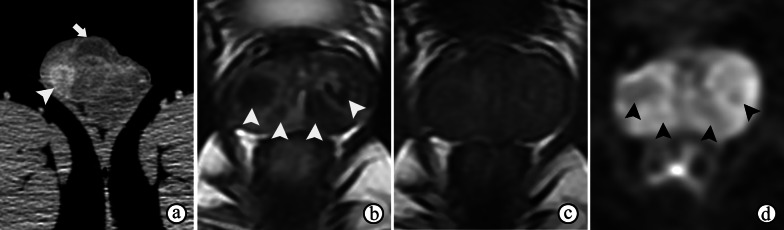
Genitourinary tuberculosis in a 26-year-old man with a right scrotal skin ulceration accompanied by pus discharge and a strongly positive tuberculin skin test. **a** Computed tomography image shows an enhanced nodule in the right epididymis (arrowhead) with testicular hydrocele (arrow). **b** T2WI shows prostate atrophy and multiple extremely hypointense nodules inside the tissue (arrowheads). **c** T2WI shows slightly inhomogeneous signal intensity of the prostate. **d** DWI at a b-value of 800 s/mm^2^ shows slight hypointensity of the nodules (arrowheads)

Tubercular involvement of the prostate gland may cause infectious granulomatous prostatitis, also known as prostate tuberculosis, which is caused by *M. tuberculosis* (via hematogenous spread or direct extension from adjacent organs). Some patients with prostate tuberculosis have a prior history of intravesical bacillus Calmette-Guérin (BCG) therapy for urothelial carcinoma [23] or present by tuberculosis in other organs of genital system (Fig. 6a) or other systems. Prostate tuberculosis is rare; only 22% of extrapulmonary tuberculosis cases affect the genitourinary system, and prostate tuberculosis is observed in only 2.7% of patients with genitourinary tuberculosis [25]. Serum PSA levels may be normal or increased. Symptoms are nonspecific, including dysuria with increased frequency and urgency.

The MRI appearances of tubercular granulomatous prostatitis are varied. Solid nodules (isolated or multifocal) may be markedly hypointense on T2WI (Fig. 6b) and infrequently hyperintense on T1WI, which may be due to the presence of paramagnetic substances, such as macrophage-laden oxygen-free radicals, in which reduce the T2 and T1 values [26]. Compared with the normal PZ, diffuse lesions usually show a diffuse, low T2 signal intensity [27] and equal or low T1 signal intensity but may be accompanied by slightly higher interior T1 signal intensity [26]. Nonnecrotic lesions may appear with moderate hyperintensity, isointensity or hypointensity on DWI (Fig. 6d). When central necrosis (caseation) occurs in tubercular granuloma, cystic areas appear. The necrosis displays various intensities on DWI, which can be hyperintense, isointense or hypointense due to differences in constituents and concentrations. Nevertheless, typical necrotic tubercular granulomatous prostatitis appears as abscesses, in which the caseation appears as hyperintensity on T2WI, hyperintensity on DWI, a low ADC, and a lack of enhancement [23]. The PI-RADS assessment may not be applicable in most cases of prostate tuberculosis.

### Prostatic neoplasms

A wide variety of tumors, both benign and malignant, occur involving the prostate. The 2016 World Health Organization classification of tumors of the prostate (Table 1) categorizes them as follows: epithelial, neuroendocrine, mesenchymal, hematolymphoid, miscellaneous and metastatic [28]. More than 95% of malignant tumors of the prostate are acinar adenocarcinomas [29]. Unusual tumors of the prostate are classified into multiple types although they constitute a small percentage of tumors.

**Table 1:** World Health Organization classification of tumors of the prostate

Category/Tumor
*Epithelial tumors*
Glandular neoplasms
Acinar adenocarcinoma
Prostatic intraepithelial neoplasia, high-grade
Intraductal carcinomac
Ductal adenocarcinoma
Urothelial carcinoma
Squamous neoplasms
Adenosquamous carcinoma
Squamous cell carcinoma
Basal cell carcinoma
*Neuroendocrine tumors*
Adenocarcinoma with neuroendocrine differentiation
Well-differentiated neuroendocrine tumor
Small cell neuroendocrine carcinoma
Large cell neuroendocrine carcinoma
*Mesenchymal tumors*
Stromal tumor of uncertain malignant potential
Stromal sarcoma
Leiomyosarcoma
Rhabdomyosarcoma
Leiomyoma
Angiosarcoma
Synovial sarcoma
Inflammatory myofibroblastic tumor
Osteosarcoma
Undifferentiated pleomorphic sarcoma
Solitary fibrous tumor
Solitary fibrous tumor, malignant
Hemangioma
Granular cell tumor
*Hematolymphoid tumors*
Diffuse large B-cell lymphoma
Chronic lymphocytic lymphoma/small lymphocytic lymphoma
Follicular lymphoma
Mantle cell lymphoma
Acute myeloid leukemia
B lymphoblastic leukemia/lymphoma
*Miscellaneous tumors*
Cystadenoma
Nephroblastoma
Rhabdoid tumor
Germ cell tumor
Clear cell adenocarcinoma
Melanoma
Paraganglioma
Neuroblastoma
*Metastatic tumors*

#### Intraductal carcinoma

Intraductal carcinoma of the prostate (IDC-P) is a newly recognized entity of epithelial tumor in the 2016 WHO classification, which is used to describe intra-acinar and/or intraductal neoplastic epithelial proliferation. Although IDC-P has some features of high-grade prostatic intraepithelial neoplasia, it presents with much greater architectural and/or cytological atypia [28]. The prevalence of IDC-P is 13–17% among radical prostatectomy (RP) specimens [30] and the incidence of IDC-P with concomitant, invasive adenocarcinoma on biopsy is less than 25%, while isolated IDC-P occurs in only less than 0.3% of biopsies [31]. The extent of IDC-P is a well-known adverse independent prognostic factor regardless of treatment and can be an important prognostic factor for the outcome [32].

**Fig. 7:**
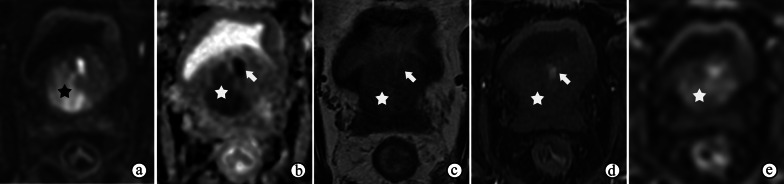
Intraductal carcinoma of the prostate in an 80-year-old man, with an elevated serum PSA level for 2 years, which was 24.46 ng/mL at the most recent measurement. **a, b** DWI at a b-value of 1400 s/mm^2^ and ADC maps show a lobulated mass with restricted diffusion in the prostate (☆). **c** Axial T2-weighted imaging shows isointensity with an ill-defined edge of the mass (☆). **d, e** Axial DCE MRI at 60 s (**d**) and 80 s (**e**) after contrast injection shows no prominent enhancement of the mass (☆). Note the hemorrhagic area in the prostate gland (arrow)

Although not assigned a Gleason grade, IDC-P is usually associated with invasive prostate carcinoma, typically with high-grade, high-stage patterns. Therefore, the MRI appearances may present as a typical csPCa of PI-RADS category > 3 with/without EPE (Fig. 7). Currin S et al. reported that the presence of intraductal spread of PCa lowers ADC values in intermediate-risk Gleason-score 7 tumors, and the PI-RADS v2 assessment category is also higher in IDC-P tumors [33].

#### Ductal adenocarcinoma

Ductal adenocarcinoma of the prostate is a rare subtype and ranks second in frequency among prostate epithelial tumors. Mixed acinar and ductal adenocarcinoma account for 5% of all PCa cases, whereas pure ductal adenocarcinoma accounts for less than 1% [34]. Ductal adenocarcinoma shows more aggressive behavior and has a worse prognosis than conventional prostatic acinar adenocarcinoma.

Ductal adenocarcinoma demonstrates similar clinical features as prostatic acinar adenocarcinoma. However, serum PSA levels may not be elevated early, as ductal adenocarcinoma originates from the ductal epithelium, which poorly secretes PSA early, only when the tumor spreads or destroys the blood-epithelial barrier.

**Fig. 8:**
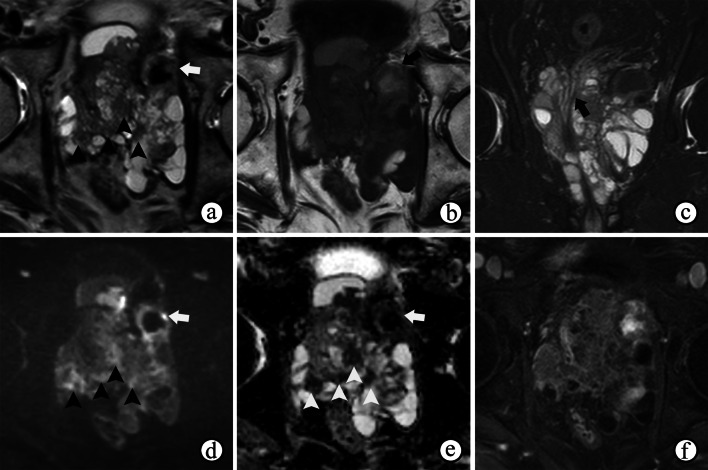
Mixed ductal and acinar adenocarcinoma in an 87-year-old man with dysuria for 12 months and a serum PSA level of 30.24 ng/mL. **a–c** Axial T2-, axial T1-, and coronal fat-suppressed T2-weighted imaging show a multilocular cyst mass occupying the prostate, periprostatic fat and bilateral seminal vesicles (black arrow on **c**), with heterogeneous signal intensity in cysts and multifocal solid areas (arrowheads on **a**), and a cyst with a thick wall and a mural nodule (arrows on **a, b**). **d, e** DWI at a b-value of 1000 s/mm^2^ and ADC maps show inhomogenously restricted diffusion of the solid areas (arrowheads) and the thick wall (arrows). **f** DCE imaging shows heterogeneous enhancement of the mass

On MRI, ductal adenocarcinoma may also present as prostatic acinar adenocarcinoma [35], and the PI-RADS assessment is applicable. However, in a few cases, ductal adenocarcinoma with/without acinar adenocarcinoma presents with a cystic/multicystic growth pattern (Fig. 8), which is due to occlusion of the prostatic duct and induction of cystic dilation, intracancerous tissue hemorrhage or central necrosis of the cancer tissue [36, 37]. The presence of cysts with thick walls or mural nodules with restricted diffusion may be malignant signs distinguishing them from cystadenoma.

#### Urothelial carcinoma

Urothelial carcinoma of the prostate can occur primarily from the prostate gland, arising from the prostatic ducts or acini and accounting for less than 5% of all prostate carcinomas [38], or synchronously with urothelial carcinoma of the bladder or urethra. It primarily manifests in middle-aged men, who are typically younger than prostatic adenocarcinoma patients. Serum PSA values for this disease are usually within the normal range.

**Fig. 9:**
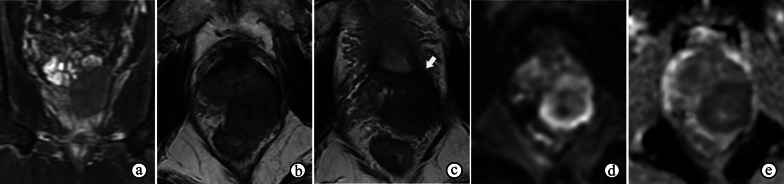
Local recurrence of urothelial carcinoma of the prostate in a 63-year-old man 1 year after completing chemotherapy. **a–c** Coronal fat-suppressed T2- (**a**) and axial T2WI (**b, c**) show an inhomogeneously isointense mass primarily located in the left lobe of the prostate and left seminal vesicle, invading a corner of the bladder (arrow on **c**). **d, e** DWI at a b-value of 1400 s/mm^2^ and ADC maps show restricted diffusion in the periphery of the mass

On MRI, urothelial carcinoma of the prostate may present as a mass similar to urothelial carcinomas of the bladder or urethra. The presence of synchronous tumors elsewhere in the genitourinary system also increases the possibility of urothelial carcinoma. The PI-RADS assessment is not suitable for use. Urothelial carcinoma exhibits a highly aggressive biological behavior and has a poor prognosis with a strong tendency of local recurrence (Fig. 9) and distant metastasis.

#### Prostatic stromal tumors

Prostatic stromal tumors arising from specialized prostate stroma have been subdivided into stromal tumors of uncertain malignant potential (STUMPs) and prostatic stromal sarcomas (PSSs), which occur at a peak incidence in the sixth and seventh decades of life [39, 40]. According to the 2016 WHO classification of tumors of the prostate, STUMPs and PSSs are classified as mesenchymal tumors [28], rather than the independent classification of prostatic stromal tumors in the 2004 WHO classification.

PSSs have been reported to affect a slightly younger population than STUMPs, and approximately half of all reported cases of PSSs occur before the age of 50 years [41, 42]. Prostatic stromal tumors are rare, and PSSs account for approximately 0.1% of primary prostate malignancies in adults [43]. The most common symptoms of presented by patients with both STUMP and PSS are chronic LUTS, but the symptoms are not specific [39, 44].

**Fig. 10:**
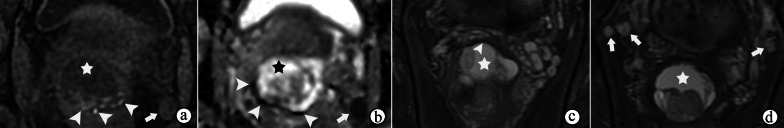
Prostatic stromal sarcoma in a 49-year-old man with a mildly elevated serum PSA level of 7.5 ng/mL. **a–c** Axial fat-suppressed T1WI, ADC maps and T2WI show a mass containing solid and necrotic components (☆). The high signal intensity area on axial fat-suppressed T1WI (**a**) and the low signal intensity area on ADC maps (**b**) both suggest a hemorrhagic change (arrowhead). **c** Coronal fat-suppressed T2WI shows an incomplete capsule around the mass and the right seminal vesicle compressed by the mass (arrowhead). **b, d** ADC maps and coronal fat-suppressed T2WI show enlarged pelvic lymph nodes (arrow)

**Fig. 11:**
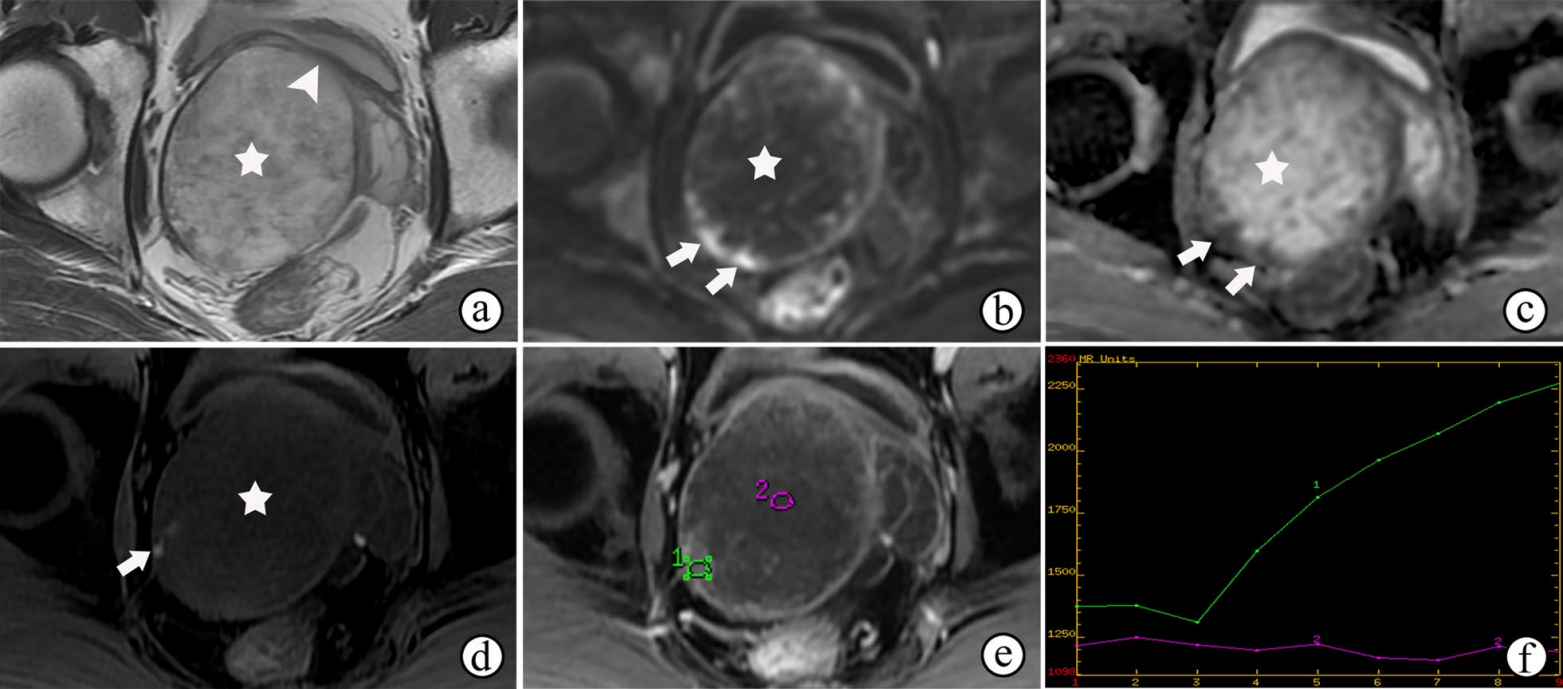
Prostatic stromal sarcoma in a 41-year-old man with a normal serum PSA level of 0.784 ng/mL. **a** Axial T2WI shows an oval mass occupying the prostate with heterogeneous hyperintensity, an incomplete hypointense capsule and compressing the bladder (arrowhead). **b, c** Axial DWI and ADC maps show cystic areas occupying the majority of the mass (☆), but containing focal solid components with remarkably restricted diffusion (arrow). **d** Axial fat-suppressed T1WI shows the mass containing a slightly high signal intensity, which suggested a hemorrhagic change (arrow). **(e–f)** Axial DCE imaging **(e)** shows that the mass had diffuse but no enhancement of the cystic components (**f, curve 2**) accompanied by a gradual moderate enhancement restricted to the solid components (**f, curve **1**)**

**Fig. 12:**
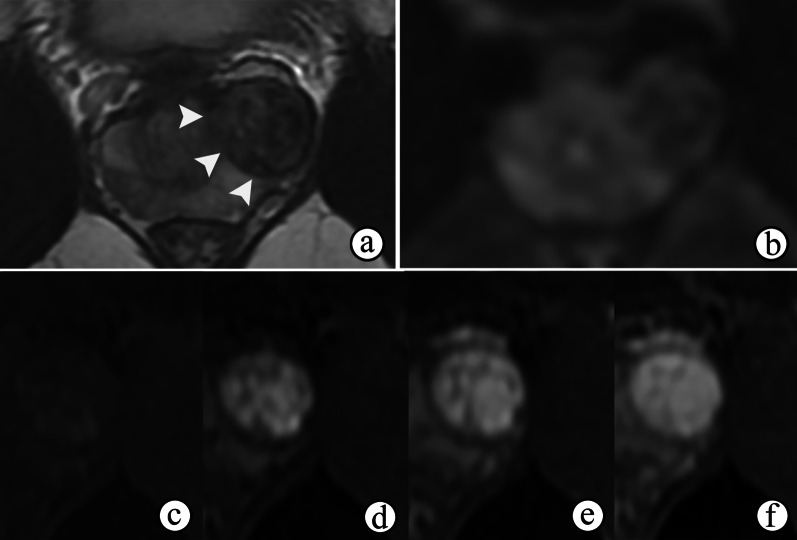
Prostate stromal tumor of uncertain malignant potential in a 39-year-old man with a normal serum PSA level of 1.780 ng/mL. **a** Axial T2WI shows a well-circumscribed nodule with heterogeneous signal and an incomplete capsule (arrowheads) located in the left PZ of the prostate and compressing the extra-prostatic tissue. **b** DWI shows hypointensity, indicating no restricted diffusion of the nodule. **c–f** Axial DCE imaging at 30 s (**c**), 60 s (**d**), 120 s (**e**) and 180 s (**f**) after contrast injection shows the early and gradual enhancement of the nodule

**Fig. 13:**
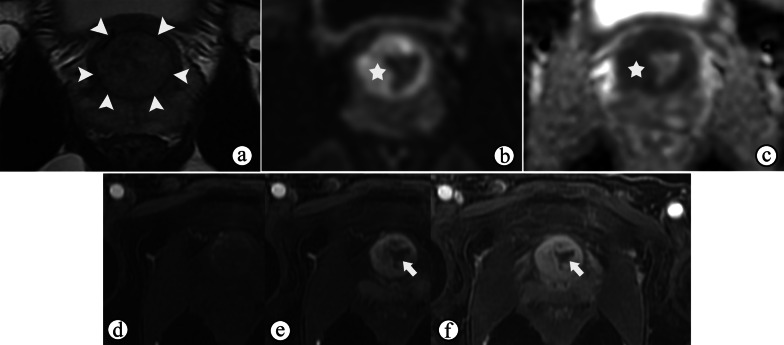
Prostate stromal tumor of uncertain malignant potential in a 33-year-old man with a normal serum PSA level of 1.080 ng/mL. **a** Axial T2WI shows a 2.7-cm nodule with heterogeneous signal in the TZ of the prostate surrounded by a capsule-like hypointense rim (arrowhead). **b, c** DWI and ADC maps show remarkably restricted diffusion of most of the nodule (☆). **d–f** Axial DCE imaging at 30 s (**d**), 60 s (**e**) and 120 s (**f**) after contrast injection shows early and gradual enhancement of the nodule containing non-enhanced areas inside the tissue (arrow)

Upon a histological examination, STUMPs and PSSs are differentiated according to the degree of cellularity and the presence of mitotic figures, necrosis, and stromal overgrowth [39]. Necrosis, cystic changes and hemorrhage are common in PSSs (Figs. 10 and 11d) because of their high malignancy and rapid growth, which may help to distinguish more aggressive PSSs from more benign variants of STUMP on MRI. MRI also helps reveal enlarged pelvic lymph nodes (Fig. 10). On MRI, the most common manifestations of STUMPs and PSSs are a rim-like hypointense “capsule” on T2WI, which is complete or incomplete (Figs. 11a, 12a and 13a). However, neither STUMPs nor PSSs contain fibrous capsules on histopathologic examination, so the term “pseudocapsule”, which represents the abrupt transition between tumor and background prostate tissue, may be more appropriate for MRI findings [5, 45]. STUMPs usually appear as cystic masses or as masses with variable amounts of cystic and solid components [5, 46]. The cystic components have been described as bloody, mucinous, or clear fluids [40]. The solid components of STUMPs usually show gradual enhancement on DCE imaging (Figs. 12, 13), which is different from the DCE wash-out of PCa. Therefore, the PI-RADS assessment may not be applicable on mpMRI of most cases of prostatic stromal tumors.

#### Prostate sarcoma

In addition to PSSs, prostate sarcomas are also mesenchymal in origin, accounting for approximately 0.1–0.2% of all primary prostate tumors [47]. Prostate sarcomas are highly aggressive tumors and usually grow rapidly. Therefore, the most common symptoms are local symptoms of the tumor’s effect on adjacent structures, such as rapid onset of urinary obstruction or a palpable mass. The serum PSA value is usually normal because of the nonepithelial origin of prostate sarcoma. At 42%, rhabdomyosarcoma is the most common prostate sarcoma, followed by leiomyosarcoma at 25%. In contrast to PCa, which primarily occurs in older people, prostate sarcoma is characterized by a wide range of onset ages. Leiomyosarcoma occurs primarily in adults between 40 and 78 years of age, while rhabdomyosarcomas are more likely to occur in children and adolescents. The principal sites of distant metastases for prostate sarcoma are the lungs, bones and liver, and bone metastases are frequently osteolytic [45], while PCa metastases primarily occur in the bone and are usually osteoblastic.

**Fig. 14:**
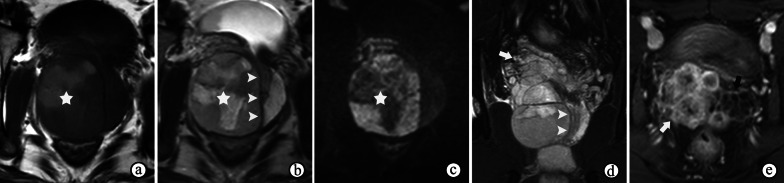
Synovial sarcoma of the prostate in a 26-year-old man, with a serum prostate-specific antigen level of 1.14 ng/mL. **a, b** T1-and T2-weighted imaging show a heterogeneous signal mass compressing the prostatic urethra and the left lobe of the prostate, with an incomplete, low T2 signal intensity pseudocapsule (arrowheads on **b**) and irregular areas of hemorrhagic and necrotic changes (☆). **c** DWI at a b-value of 1200 s/mm^2^ shows a high signal intensity of the solid area and low signal intensity of the cystic area of the mass (☆). **d** Coronal fat-suppressed T2WI shows the mass compressing the prostatic urethra and the left lobe of the prostate (arrowheads), compressing and invading the right seminal vesicle (arrow). **e** DCE imaging shows heterogeneous enhancement of the lobulated mass invading seminal vesicles (white arrow). Note the residual tissue of the left seminal vesicle (black arrow)

Most prostate sarcomas appear as large masses with hypointensity on T1WI and heterogeneous isointensity and hyperintensity on T2WI. Hemorrhagic and necrotic changes are common because of their high malignancy and rapid growth (Figs. 10a, b, 14a, b). Despite their locally aggressive features, prostate sarcomas often compress adjacent tissues or organs, as opposed to solely directly invading them [45], with a complete or incomplete and low T2 signal intensity for the compressible pseudocapsule (Figs. 10, 11 and 14). Prostate sarcomas are rarely confused with PCa, which usually extends by infiltrating its adjacent tissues, so it is commonly ill-defined without a capsule on T2WI. However, leiomyosarcomas and alveolar rhabdomyosarcomas are more infiltrative than other forms of prostate sarcomas, perhaps with poorly defined tumor margins on MRI [5]. Although PI-RADS is not applicable for prostate sarcomas, mpMRI helps to determine the site of origin of the tumor, its local extent, and its tissue characteristics and aids in planning surgical resection or adjunctive therapy.

#### Solitary fibrous tumor

A solitary fibrous tumor (SFT) is a mesenchymal tumor derived from CD34-positive mesenchymal cells and was first described in the pleura [48]. Extrapleural SFTs account for approximately 0.6% of the total incidence of soft tissue tumors [49]. Malignant SFTs of the prostate are extremely rare [50, 51].

SFTs of the prostate are slow-growing tumors usually seen among middle-aged and elderly individuals. The most frequent symptoms are obstructive urinary symptoms caused by a progressive increase in the size of the tumor [52]. The tumor dimensions are quite variable, ranging from 2 to 18 cm, with many reported to be > 5 cm [53, 54]. SFTs located in the prostate may originate from the prostate or prostatic urethra [55].

**Fig. 15:**
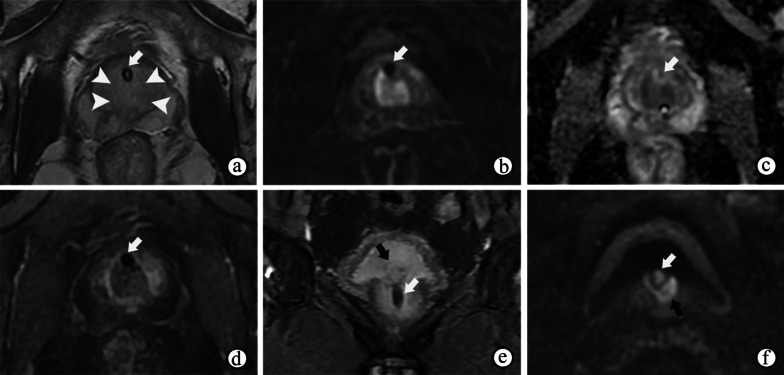
Malignant solitary fibrous tumor originating from the prostatic urethra in a 62-year-old male with a slightly elevated serum PSA level (4.770 ng/mL) and a Foley catheter to relieve urinary retention (white arrow). **a** T2WI shows a heterogeneously hyperintense nodule (1.8 cm in size) with an obscured hypointense capsule (arrowhead) behind and adjacent to the prostatic urethra in the prostate. **b, c** DWI at a b-value of 1400 s/mm^2^ and ADC maps show that the nodule exhibits mildly and inhomogeneously restricted diffusion. **d** DCE imaging shows continuously mild and heterogeneous enhancement of the nodule. **e, f** Coronal fat-suppressed T2WI and DWI at a b-value of 1400 s/mm^2^ show the nodule protruding into the bladder (black arrow)

SFTs generally appear as well-circumscribed masses on MRI (Fig. 15), with heterogeneous intensity on T2WI. The variable signal intensity on T2WI depends mainly on areas of collagen and fibroblasts, vascular and hypercellular areas, and the presence of degeneration [56]. Both of these appearances differ from those of PCa, which are poorly circumscribed on MRI and homogeneously hypointense on T2WI. In addition, SFTs exhibit more moderately restricted diffusion on DWI and ADC maps and more moderate enhancement on DCE imaging than PCa [57]. Therefore, although SFTs have few distinctive features on mpMRI, they can be distinguished from PCa, and the PI-RADS assessment may not be suitable for use.

The characteristics of malignant extrapleural SFTs include a large tumor size (greater than 10 cm in diameter), hypercellularity, nuclear atypia, a high mitotic index (> 4 mitoses/10 high-power fields), necrosis and infiltrative margins [58]. Considering the rarity of SFTs of the prostate and the low predictability of their malignant potential [59], long-term clinical follow-up is recommended.

#### Lymphoma

Malignant lymphoma involving the prostate is a rare kind of extranodal lymphoma, and diffuse large B cell lymphoma (DLBCL) is the most frequently seen type. The prostate gland is the site of 0.1% of non-Hodgkin lymphomas (NHLs) and 0.9% of extranodal lymphomas. Primary malignant lymphoma involving the prostate, which is located in the prostate without evidence of systemic lymph node invasion by lymphoma cells, accounts for 0.09% of all prostatic malignancies [60], and is less common than secondary lymphoma. The serum PSA level of prostatic lymphoma is usually less than 4 ng/mL. In addition to LUTS, systemic symptoms of lymphoma may be present, although uncommon, including fever, chills, night sweats, and weight loss [61, 62].

**Fig. 16:**
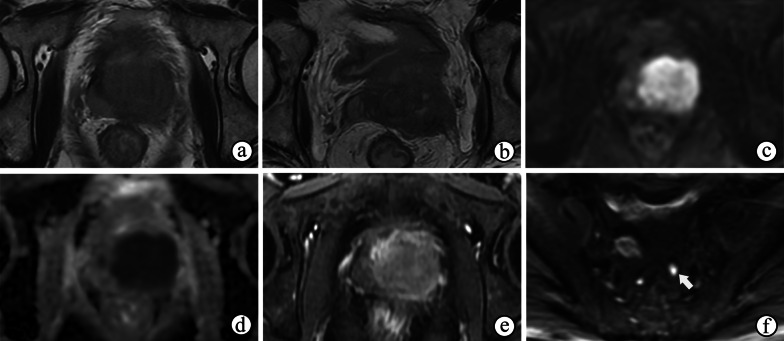
Secondary diffuse large B cell lymphoma involving the prostate in a 66-year-old male with a normal serum prostate-specific antigen level (0.795 ng/mL) and tear-like pain in the lower abdomen for 1 month. **a, b** T2WI shows a homogeneously isointense mass in the prostate with extraprostatic extension and invasion of the seminal vesicles. **c, d** DWI at a b-value of 1400 s/mm^2^ and ADC maps show that the mass exhibits observably restricted diffusion. **e** DCE imaging shows moderate enhancement of the mass. **f** DWI at a b-value of 800 s/mm^2^ shows slightly enlarged pelvic lymph nodes

Prostatic lymphomas grow rapidly and invade surrounding organs easily; therefore, they often appear as large, irregular and lobulated masses with or without invasion of neighboring organs on prostate MRIs (Fig. 16b), and the PI-RADS assessment may be used unavoidably for prostatic lymphomas. They are usually homogeneously isointense on T1WI/T2WI and present with homogeneously restricted diffusion on DWI and ADC maps (Fig. 16) because they are rarely accompanied by hemorrhage and necrosis [63]. On DWI and ADC maps, lymphomas show observably restricted diffusion as a result of the high density of lymphoma cells, their high karyoplasmic ratio, abundant extracellular microstructures and intracellular proteins. On DCE imaging, moderate enhancement of the mass may be observed [64].

#### Prostatic cystadenoma

Prostatic cystadenoma is a rare benign tumor consisting of cysts and prostatic glands. Aa large range for the age at the time of presentation has been reported, from 23 to 80 years, and the most frequent clinical symptoms are LUTS [65]. The serum PSA level is often, but not always, elevated, as it is expressed by the neoplastic tissue, consisting of dilated glandular structures and prostatic epithelia [66].

**Fig. 17:**
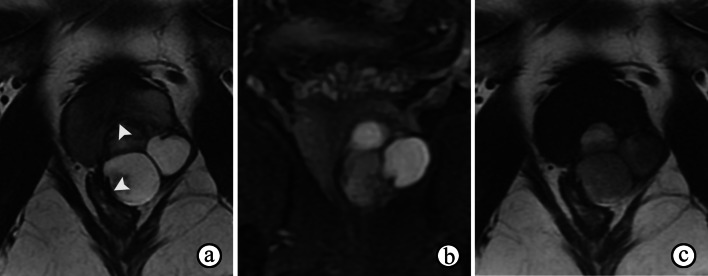
Prostatic cystadenoma in a 36-year-old man. **a, b** Axial T2- and coronal fat-suppressed T2WI show a multilocular cystic mass with inhomogeneously high signal intensity from the left lobe of prostate, compressing the tissue of the prostate and rectum (arrowheads on **a**). **c** Axial T1WI shows inhomogeneous hyperintensity of the cysts suggesting hemorrhage or proteinaceous fluid

The typical MR futures of prostatic cystadenomas are easy to identify, and the PI-RADS assessment is not applicable. Prostatic cystadenomas can grow to large proportions, causing distinct mass effects but not invading adjacent tissues. On T2WI, prostatic cystadenomas appear as multilocular cystic lesions of high signal intensity with surrounding isointense soft tissue. Variations in T2 and T1 signal intensities are suggestive of hemorrhage or proteinaceous fluid (Fig. 17). Occasionally, the presence of fluid–fluid levels may occur [47]. ADC maps of the lesions usually show no restricted diffusion in the cysts but lower ADC values in the septa and solid components. In addition, enhancement of the septa and solid components may be observed.

## Conclusions

The clinical MRI diagnostic workflow for prostatic diseases listed below is recommended when clinicians are deciding whether to use the PI-RADS assessment.

First, past relevant history and recent symptoms should be considered. Iatrogenic causes (after surgical operation or BCG therapy for urothelial carcinoma) and allergy may result in granulomatous prostatitis (including tuberculosis). In addition to nonspecific LUTS, some symptoms and signs may provide more specific information. Prostate tuberculosis may be accompanied by active tuberculosis in other organs or systems. Fever, chills, urinary frequency and urgency, suprapubic pain, and pus cells on urine examination are typical for abscesses. Systemic symptoms of lymphoma include fever, chills, night sweats, weight loss or lymphoma in other tissues. Rapid onset of urinary obstruction or a palpable mass may hint at tumors with rapid growth or invading the prostatic urethra.

Second, age and serum PSA levels should be taken into account because they are important factors in differential diagnoses. PCa and BPH tend to occur in individuals older than 50 years with elevated serum PSA levels, while serum PSA levels in patients with other tumors are usually in the normal range, except for prostatic cystadenoma because its neoplastic tissue expresses PSA. Some malignant mesenchymal tumors, such as rhabdomyosarcoma, are more common in children and adolescents with normal serum PSA levels. In addition, the serum PSA levels of granulomatous prostatitis may be elevated or normal due to the progression of or improvement in the disease.

Third, noninvasive mpMRI provides comprehensive information that is needed for preoperative diagnosis and evaluation of prostate diseases. Granulomatous prostatitis and lymphoma mimic PCa on mpMRI. BPH nodules outside the TZ are analogous to those in the TZ on mpMRI, and the size of the nodule may also increase with the progression of BPH in the TZ. Prostate abscesses demonstrate central necrosis in the interior inside presenting with hyperintensity on T2WI, hyperintensity on high b-value DWI, and low ADC, with rim enhancement in the exterior. Malignant mesenchymal tumors usually exhibit hemorrhagic, cystic or necrotic changes with complete or incomplete capsules and cause distinct mass effects.

By combining the above three steps, it is possible to decide whether to use PI-RADS assessment. PI-RADS assessments are suggested for abnormities simulating PCa, and biopsy should be considered for PI-RADS category 4 or 5. For diseases unsuitable for PI-RADS assessment, it is important to draw a few qualitative conclusions or primarily differentiate the pathological condition if possible. However, biopsy may also be necessary for PI-RADS 1–3 lesions in patients with very high serum PSA levels. In conclusion, although the detection and staging of PCa are the most common tasks when performing prostate mpMRI and using the PI-RADS assessment, radiologists must continue to consider that not every prostate lesion is an adenocarcinoma and must have an intimate knowledge of other diseases. Past relevant history, symptoms, age, serum PSA levels, mpMRI manifestations, and the applicability of PI-RADS assessment (Table 2) should be considered to reduce unnecessary biopsy while maintaining the diagnostic sensitivity for malignant lesions.

**Table 2:** Primary clinical or mpMRI characteristics of prostatic abnormalities

Prostatic abnormalities	Primary clinical or mpMRI characteristics
Acinar adenocarcinoma with/without other glandular neoplasms	Age: elderly men PSA: sharply elevated PI-RADS: suitable
BPH	Age: elderly men PSA: slightly elevated PI-RADS: suitable
Chronic prostatitis	PSA: slightly elevated PI-RADS: suitable
Benign hyperplastic nodules outside the TZ	PSA: Slightly elevated PI-RADS: unsuitable
Granulomatous prostatitis	Past relevant history: iatrogenic causes (after surgical operations or BCG therapy for urothelial carcinoma) and allergy PSA: transiently elevated PI-RADS: unavoidable
Prostate tuberculosis	Past relevant history: BCG therapy for urothelial carcinoma Symptoms: active tuberculosis in other organs or systems PSA: transiently elevated PI-RADS: unsuitable
Abscess	Symptoms: fever, chills, urinary frequency and urgency, suprapubic pain, and pus cells on urine examination PSA: transiently elevated mpMRI findings: central necrosis in the interior presenting with hyperintensity on T2WI, hyperintensity on high b-value DWI, and low ADC, with rim enhancement in the exterior PI-RADS: unsuitable
Mesenchymal tumors	Age: younger men PSA: normal mpMRI findings: hemorrhagic, cystic or necrotic changes with complete or incomplete capsule causing distinct mass effects PI-RADS: unsuitable
Lymphoma	PSA: normal Symptoms: LUTS, systemic symptoms (including fever, chills, night sweats, and weight loss) PI-RADS: unavoidable
Prostatic cystadenoma	Age: large age range mpMRI findings: cystic lesions PI-RADS: unsuitable

*PSA* prostate-specific antigen, *PI-RADS* Prostate Imaging Reporting and Data System, *BPH* benign prostatic hyperplasia, *TZ* transitional zone, *BCG* bacillus Calmette–Guérin, *mpMRI* multiparametric magnetic resonance imaging, *T2WI* T2-weighted imaging, *DWI* diffusion-weighted imaging, *ADC* apparent diffusion coefficient, *LUTS* lower urinary tract symptoms

## Data Availability

The data of cases in the manuscript are available from the corresponding author on reasonable request.

## Abbreviations

ADC: Apparent diffusion coefficient
BCG: Bacillus Calmette-Guérin
BPH: Benign prostatic hyperplasia
csPCa: Clinically significant prostate cancer
CZ: Central zone
DCE: Dynamic contrast-enhanced
DLBCL: Diffuse large B cell lymphoma
DWI: Diffusion-weighted imaging
EPE: Extraprostatic extensions
IDC-P: Intraductal carcinoma of the prostate
LUTS: Lower urinary tract symptoms
mpMRI: Multiparametric magnetic resonance imaging
NHL: Non-Hodgkin lymphoma
PI-RADS: Prostate Imaging Reporting and Data System
PSA: Prostate-specific antigen
PSS: Prostatic stromal sarcoma
PZ: Peripheral zone
RP: Radical Prostatectomy
SFT: Solitary fibrous tumor
STUMP: Stromal tumor of uncertain malignant potential
T2WI: T2-weighted imaging
TURP: Transurethral resection of prostate
TZ: Transitional zone
